# Necrology

**Published:** 1898-11

**Authors:** 


### NECROLOGY.

Thomas Giffen, M.R.C.V.S., died at his home, 217 West Fifty-eighth Street, New York City, in August, after a lingering illness. Deceased was a member of the U. S. V. M. A. and New York State Society, and at one time a member of the New York County Association.

John P. Messer, D.V.S., graduate of the American Veterinary College, class of '97, died at his home in New York City, September 1st, from fever contracted in the recent war against the Spanish.

LaForest E. Turner, D.V.S., formerly of New York City, but who had been holding a position in the Seventh Cavalry, U. S. A., died at Fort Grant, Arizona, September 21st, of congestion of the brain. Deceased was a graduate of the American Veterinary College, 1891.

Frederick William Turner, D.V.S., at 91 Lawrence Street, New York City, in September. Graduate of the American Veterinary College, class of '90, and a member of the U. S. V. M. A. and New York County Veterinary Medical Association.

William Machan, V.S., graduate of Ontario Veterinary College, class of '94, died at his home, 358 West Forty-eighth Street, New York City, October 11th, from meningitis, caused by the fall of a horse, crushing his skull. Deceased was a member of the New York State and New York County Veterinary Medical Associations.

E. Bovette, V.S., of Denver, Colorado, lost his life in October while fishing in one of the lakes some ten miles from that city. Dr. Bovette came of a family with numerous representatives in the veterinary profession. He enjoyed a lucrative practice in Denver; was active in the Colorado State Association and officiated as Veterinarian to the City Troop of Denver.

